# Bibliometric analysis of research trends in the relationship between frailty and neoplasms over the past decade

**DOI:** 10.1007/s00520-024-08744-4

**Published:** 2024-07-23

**Authors:** Yuqin Chen, Xiaoping Chen, Lifang Zhong, Huiming Lu, Huiting Zhang, Mengxiao Jiang

**Affiliations:** 1 Department of Urology, State Key Laboratory of Oncology in South China, Guangdong Provincial Clinical Research Center for Cancer, Sun Yat-Sen University Cancer Center, Guangzhou, 510060 P. R. China; 2 Department of Breast Cancer, State Key Laboratory of Oncology in South China, Guangdong Provincial Clinical Research Center for Cancer, Sun Yat-Sen University Cancer Center, Guangzhou, 510060 P. R. China

**Keywords:** Frailty, Neoplasms, Bibliometric, VOSviewer, CiteSpace

## Abstract

**Background:**

The relationship between frailty and neoplasms has attracted increasing attention from researchers in recent years. This study aims to identify current research hotspots and status in this field through bibliometric and visualization analysis.

**Methods:**

Literature on the relationship between frailty and neoplasms, meeting the inclusion criteria, was collected from the Core Collection. Bibliometric analysis and visualization were performed using WoS, VOSviewer, and CiteSpace.

**Results:**

Our study included 7410 documents on frailty and neoplasms, authored by 43,605 researchers from 9478 institutions across 115 countries, and published in 2067 journals. The USA emerged as the most productive and influential country in this field, with 3059 publications and 89,319 citations. The University of Texas MD Anderson Cancer Center and Mayo Clinic were recognized as the most productive institution and the institution with the highest citation count, respectively. The *Journal of Geriatric Oncology* was the leading publisher. Kirsten K Ness and James L Kirkland were identified as the most productive and most cited authors, respectively. Cluster analysis identified five key areas: body condition and nutrition, quality of life, frailty, mortality and care, and the elderly and frailty.

**Conclusion:**

The relationship between frailty and neoplasms remains a contentious and frequently discussed topic. Our findings indicate that research primarily focuses on cancer, the elderly, clinical trials, adverse health outcomes, frailty assessment, and nutrition.

Identified as early as 1966, frailty is one of the most common geriatric syndromes. Scholars have been investigating its causes and treatments. In 2007, research indicated that vitamin D deficiency may lead to changes in bone health [1], which could subsequently cause frailty [2]. However, it was later discovered that vitamin deficiency is not a major cause of frailty, except possibly in a small percentage of geriatric patients [3]. Frailty is currently defined as a state of heightened vulnerability to stressors due to declines in function and reserves across various physiologic systems. This condition is characterized by muscle weakness, fatigue, slowed motor performance, low physical activity, and unintentional weight loss [4].

Frailty is prevalent among cancer patients, particularly in older individuals. Reports suggest that the prevalence of frailty in elderly cancer patients ranges from 40 to 50%, and it can reach up to 90% in specific groups [5]. Frailty impacts patients’ quality of life and treatment tolerance and significantly increases the risk of treatment-related complications and mortality [6].

In recent years, surgeons have increasingly focused on the damage and complications from surgical procedures, with frailty emerging as a significant factor, particularly in the rehabilitation of cancer patients post-surgery [7]. Frailty, a complex and multidimensional state, is marked by reduced physiological reserves, leading to decreased resilience, impaired adaptive capacity, and heightened vulnerability to stressors [8]. Factors such as vulnerability to stressors, reduced physiological reserves, malnutrition, disability, and physical deconditioning can affect decision-making strategies, impact treatment completion, and elevate toxicity risk [9]. Digestive system cancer often leads to malnutrition, prompting considerable research into the relationship between frailty and affected patients [10]. However, the relationships between sarcopenia and surgery, as well as the underlying mechanisms, remain not fully understood. Understanding the role of sarcopenia in surgical procedures could improve patients’ quality of life and accelerate post-surgery rehabilitation.

Recent years have seen a surge in studies exploring the relationship between neoplasms and frailty [11, 12]. The surge in publications could overwhelm researchers interested in this relationship, making it challenging to stay at the forefront of this field. As a discipline within literature and information science, bibliometric analysis can identify publication characteristics and assess study trends through qualitative and quantitative methods [13]. Using software for clustering and other analyses, bibliometrics can help identify research topics from generated clusters and systematically organize vast amounts of information after multiple data analyses [14]. Bibliometric analyses in this field have been scarce. This study aims to offer researchers insights into the current structure and development of studies on the frailty-neoplasms relationship through bibliometric and visual analysis. We collected and summarized studies published from 2014 to 2023 on the neoplasms-frailty relationship, analyzing and visualizing their knowledge structure and research trends.

## Materials and methods

### Data source

The Web of Science (WoS, Clarivate Analytics, Philadelphia, PA, USA) ranks among the most extensive scientific databases, frequently utilized in bibliometric analyses [15]. The Web of Science Core Collection (WoSCC) comprises leading scholarly journals, books, and conference proceedings across sciences, social sciences, arts, and humanities, offering a comprehensive citation network. To minimize potential biases from article updates, two independent researchers jointly conducted the search, concluding it on January 22, 2024.

### Search strategies

Search terms for frailty included increased vulnerability to stressors, declines in function and reserves across multiple physiologic systems, muscle weakness, fatigue, slowed motor performance, low physical activity, unintentional weight loss, frailty, and lack or loss of strength and energy. Neoplasms search terms included new abnormal tissue growth, with malignant neoplasms characterized by a higher degree of anaplasia and properties of invasion and metastasis, compared to benign neoplasms. The search formula is presented as follows: TS = (neoplasm* OR CANCER* OR CARCINOMA OR TUMOR*) AND (Asthenia OR Frailties OR Frailness OR “Frailty Syndrome” OR Debilit*).

### Retrieval strategies

Inclusion criteria for this study were (a) articles examining the frailty-neoplasms relationship; (b) documents classified as articles or reviews; and (c) publications dated between January 1, 2014, and December 31, 2023. Exclusion criteria included (a) non-English publications and (b) documents lacking sufficient information. Studies that met the inclusion criteria and did not fall under the exclusion criteria were included in this study.

### Documents extraction

The full records of the identified documents were extracted from the WoS database. The information contained in the records included title, authors, keywords, journals, abstract, year of publication, countries/regions, affiliations, research direction, and funding agencies.

### Bibliometric and visualized analysis

A predicted publication curve, based on annual publication counts, was generated. Network maps showcasing collaborative and citation relationships among countries, institutes, and authors were created using VOSviewer [16] version 1.6.19 (Leiden University Center for Science and Technology Studies, Leiden, the Netherlands) and CiteSpace 6.2.R3 [17]. In the VOSviewer-generated maps, various nodes represent different entities, including authors and countries. Lines between nodes indicate the relationships among entities, while Total Link Strength (TLS) quantifies the connection strength between nodes [18]. We conducted burst detection on keywords and literature and used the timeline method for visual analysis of the keywords.

## Results

### Overview information and trends of publications

**Fig. 1:**
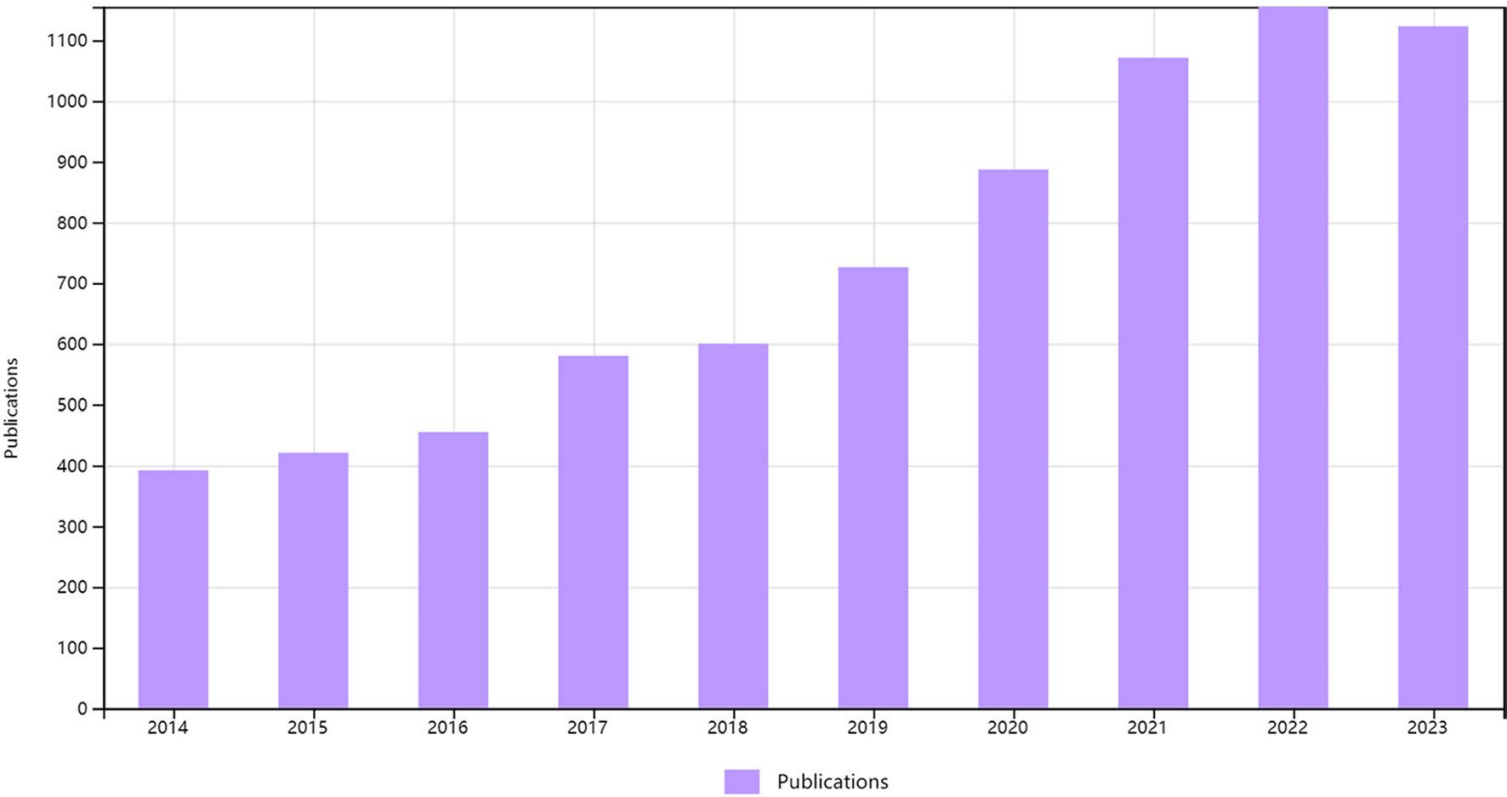
The publication number in the past 10 years

Our study included 7410 documents exploring the frailty-neoplasms relationship. These articles were authored by 43,605 individuals from 9478 institutions across 115 countries, appearing in 2067 journals. Between 2014 and 2023, publications in this field saw rapid growth. In 2014, 392 articles were published, with the number significantly increasing in subsequent years. Since 2017, over 500 studies were published annually, surpassing 1000 in 2021, and exceeding 1100 just a year later (Fig. 1).

### Analysis of countries or regions

**Fig. 2:**
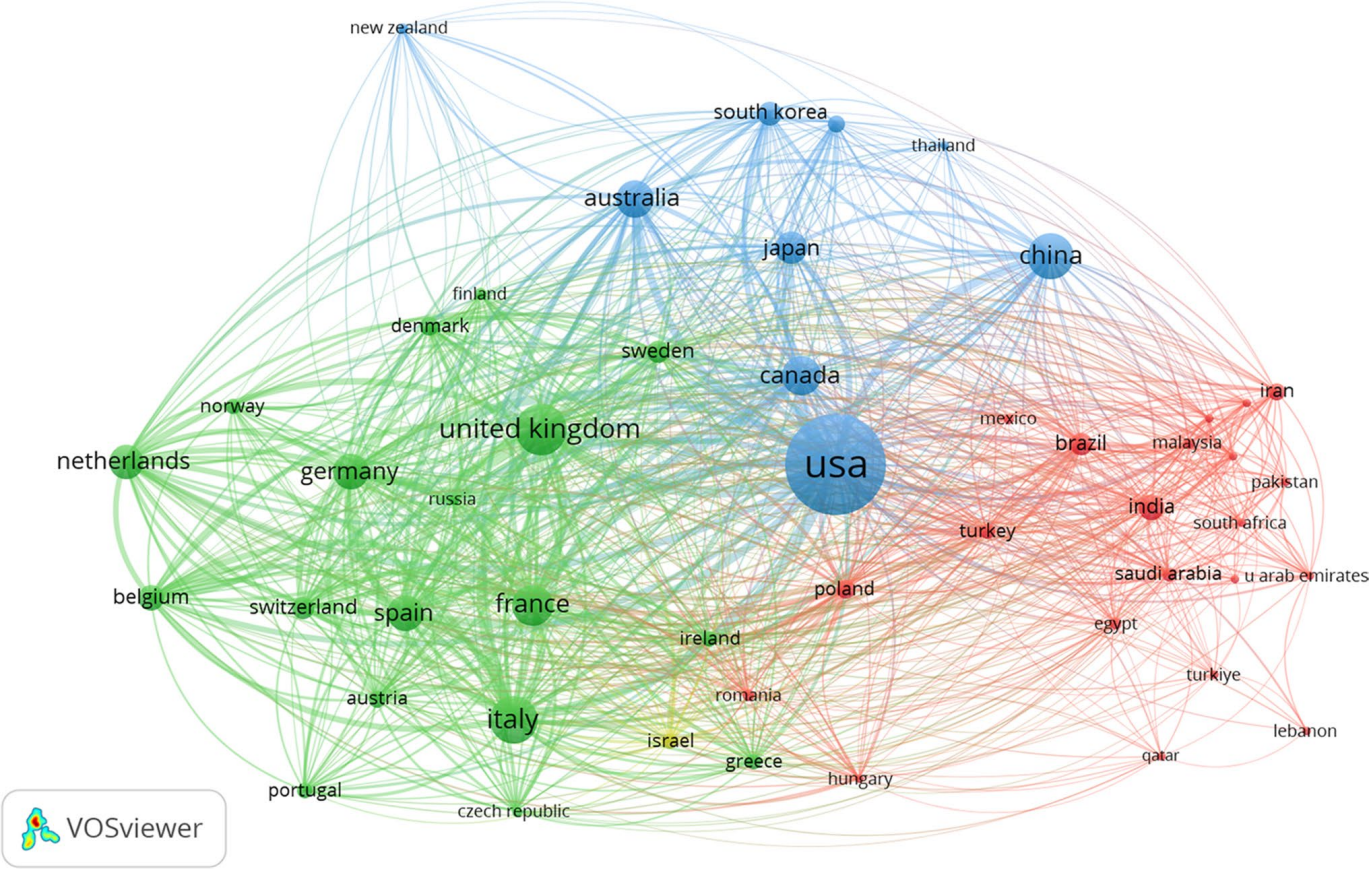
Map of collaboration relationships between countries

**Fig. 3:**
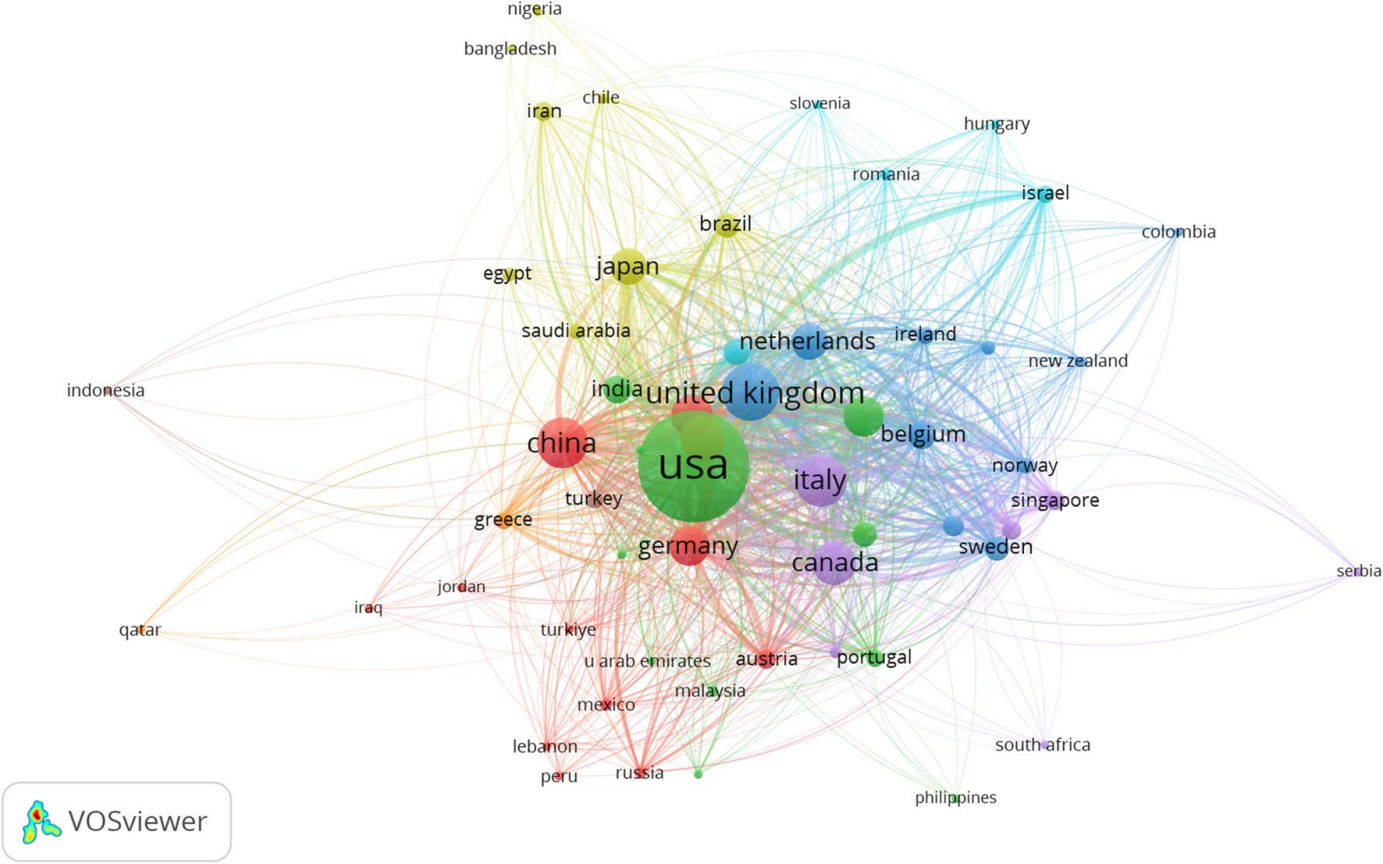
Map of citation relationships between countries

Table 1 summarizes the publication output of the top 10 most productive countries. The USA, leading in both documents and citations, dominates the field, significantly outpacing other countries. European countries, with the UK at the forefront, were the next most productive. Undoubtedly, the USA was the most influential country in studying the relationship between frailty and neoplasms, owing to its highest number of publications and citations. Figure 2 visualizes the collaboration relationships between countries. In this map, colored shapes indicate each country’s publication count, while lines denote inter-country collaborations. Over the past decade, the USA and European countries have been the most frequent collaborators. Figure 3 displays the citation relationships between countries. Node size corresponds to each country’s publication count, and the color of lines between two nodes indicates citation relationships between countries. Thicker lines between nodes signify a higher frequency of mutual citations. The USA, Italy, the UK, and the Netherlands exhibited the most frequent citation relationships.

**Table 1:** The top 10 countries with the largest number of publications on the relationship between frailty and neoplasms

Country	Documents	Citations	TLS
USA	3059	89,319	2214
UK	812	29,299	1556
Italy	693	26,057	1358
France	529	23,276	1254
Canada	494	17,370	851
Germany	403	16,649	1195
Spain	391	16,029	1077
Australia	445	15,666	684
Netherlands	354	13,761	703
China	645	13,744	539

### Analysis of institutions

**Fig. 4:**
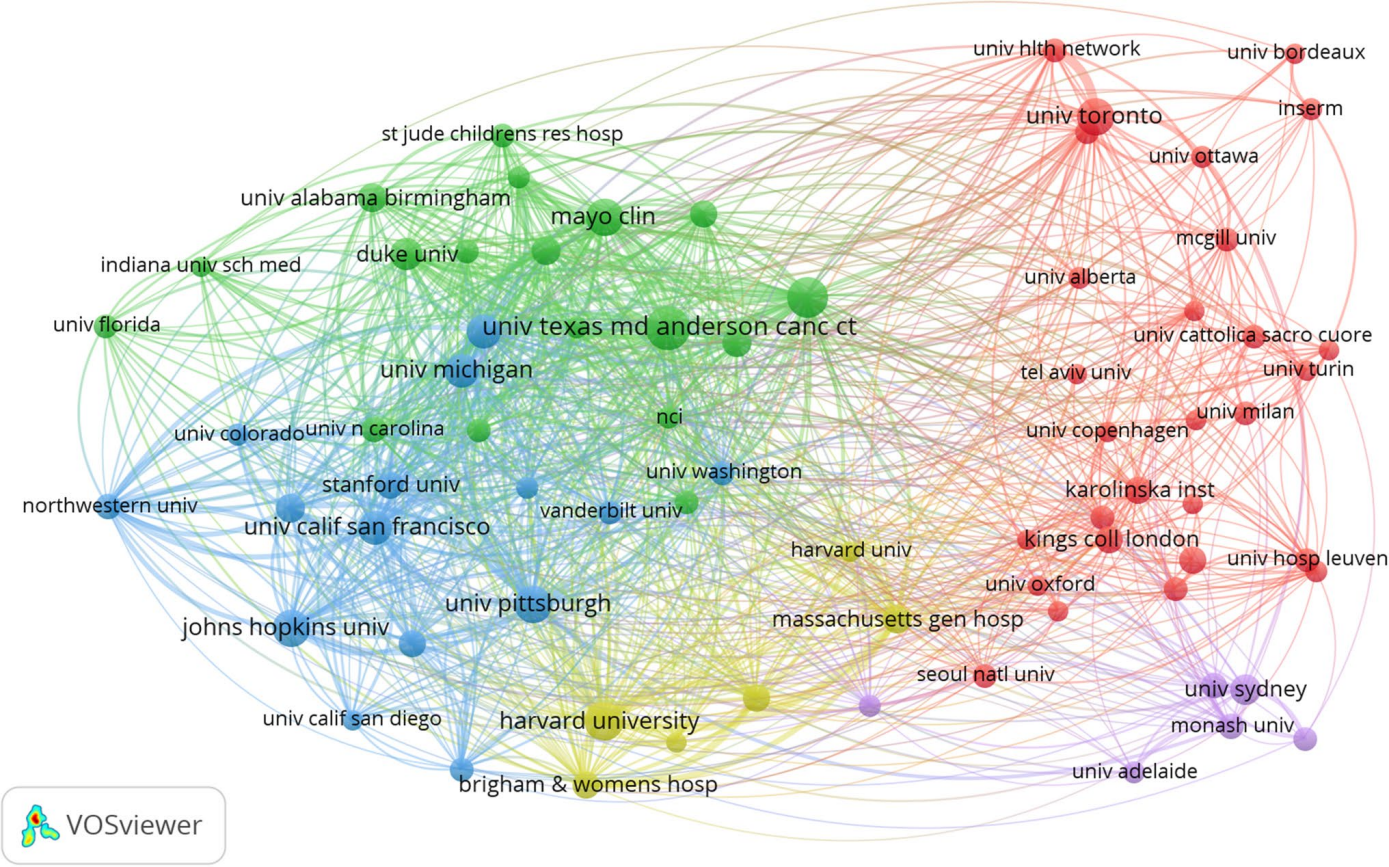
Map of collaborative relationships between institutions

**Fig. 5:**
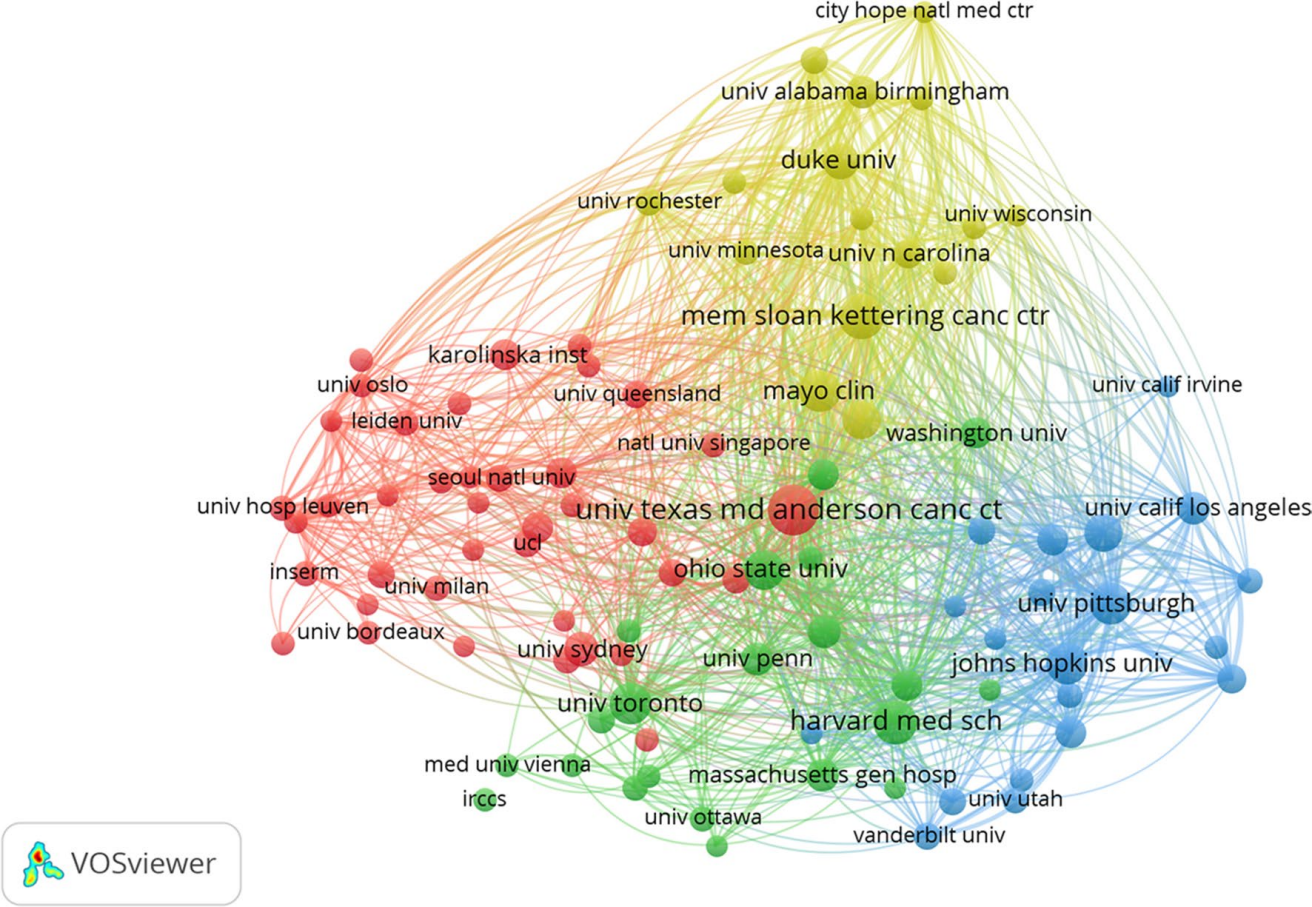
Map of citation relationships between institutions

A total of 9478 institutions conducted research on the frailty-neoplasms relationship. Table 2 summarizes the top 10 institutions by publication count. With 182 publications, the University of Texas MD Anderson Cancer Center ranked as the most productive institution in this field. Figure 4 visualizes the collaborative relationships between institutions. The Memorial Sloan Kettering Cancer Center, the University of Pittsburgh, and Harvard Medical School, with the highest TLS, were central in the collaboration map. Figure 5 displays the citation relationships among institutions. Node size indicates the number of publications, while lines represent citation relationships. The Mayo Clinic received the highest number of citations (cited times = 7408).

**Table 2:** The top 10 institutions with the largest number of publications on the relationship between frailty and neoplasms

Organization	Documents	Citations	TLS
University of Texas MD Anderson Cancer Center	182	5860	500
Memorial Sloan Kettering Cancer Center	148	4050	569
Harvard Medical School	142	3062	512
University of Toronto	131	2096	422
Johns Hopkins University	128	3433	343
University of Pittsburgh	127	3511	558
Mayo Clinic	124	7408	348
Ohio State University	111	4895	407
University of Michigan	105	2425	310
University of California, San Francisco	103	3130	432

### Analysis of journals

**Fig. 6:**
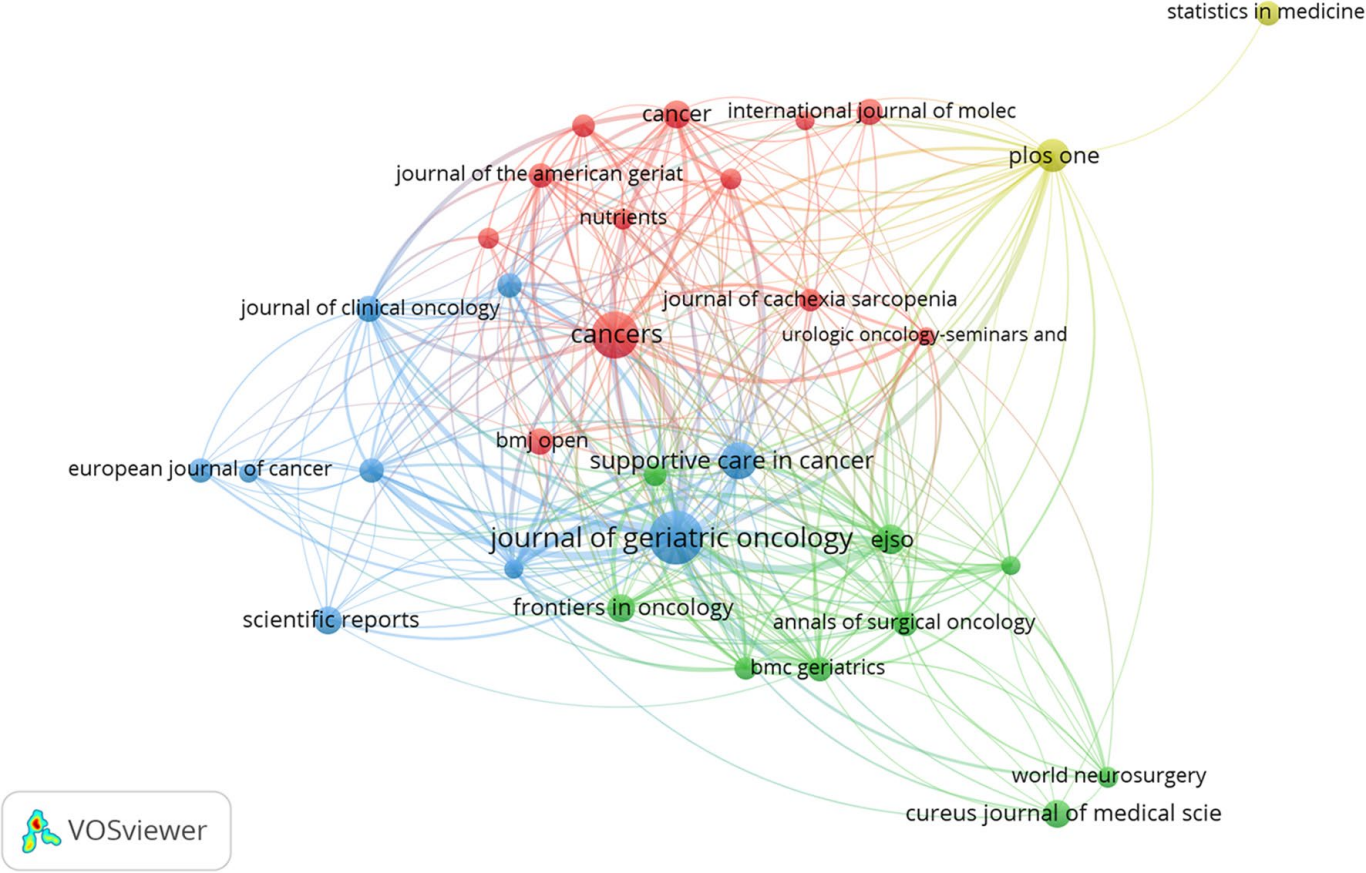
Map of cited relationships between journals

A total of 2067 journals have published research on the frailty-neoplasms relationship. Table 3 ranks the top 10 journals by the number of studies published in this field. The *Journal of Geriatric Oncology* (207 publications) and *Cancers* (159 publications) each published over 100 studies. *Lancet Oncology* was the most cited journal, with 6504 citations. Figure 6 depicts the citation relationships among journals. *Lancet Oncology*, *Journal of Clinical Oncology*, and *Journal of Geriatric Oncology* were the most cited journals in this field.

**Table 3:** The top 10 journals with the largest number of publications on the relationship between frailty and neoplasms

Journal	Documents	Citations	TLS
*Journal of Geriatric Oncology*	207	2746	1262
*Cancers*	159	1085	446
*Supportive Care in Cancer*	93	1512	240
*PloS One*	77	1796	182
*EJSO*	67	1179	424
*Frontiers in Oncology*	60	359	167
*Cureus Journal of Medical Science*	56	78	35
*Cancer*	55	1615	308
*Scientific Reports*	54	793	41
*BMJ Open*	52	339	75

### Analysis of authors

**Fig. 7:**
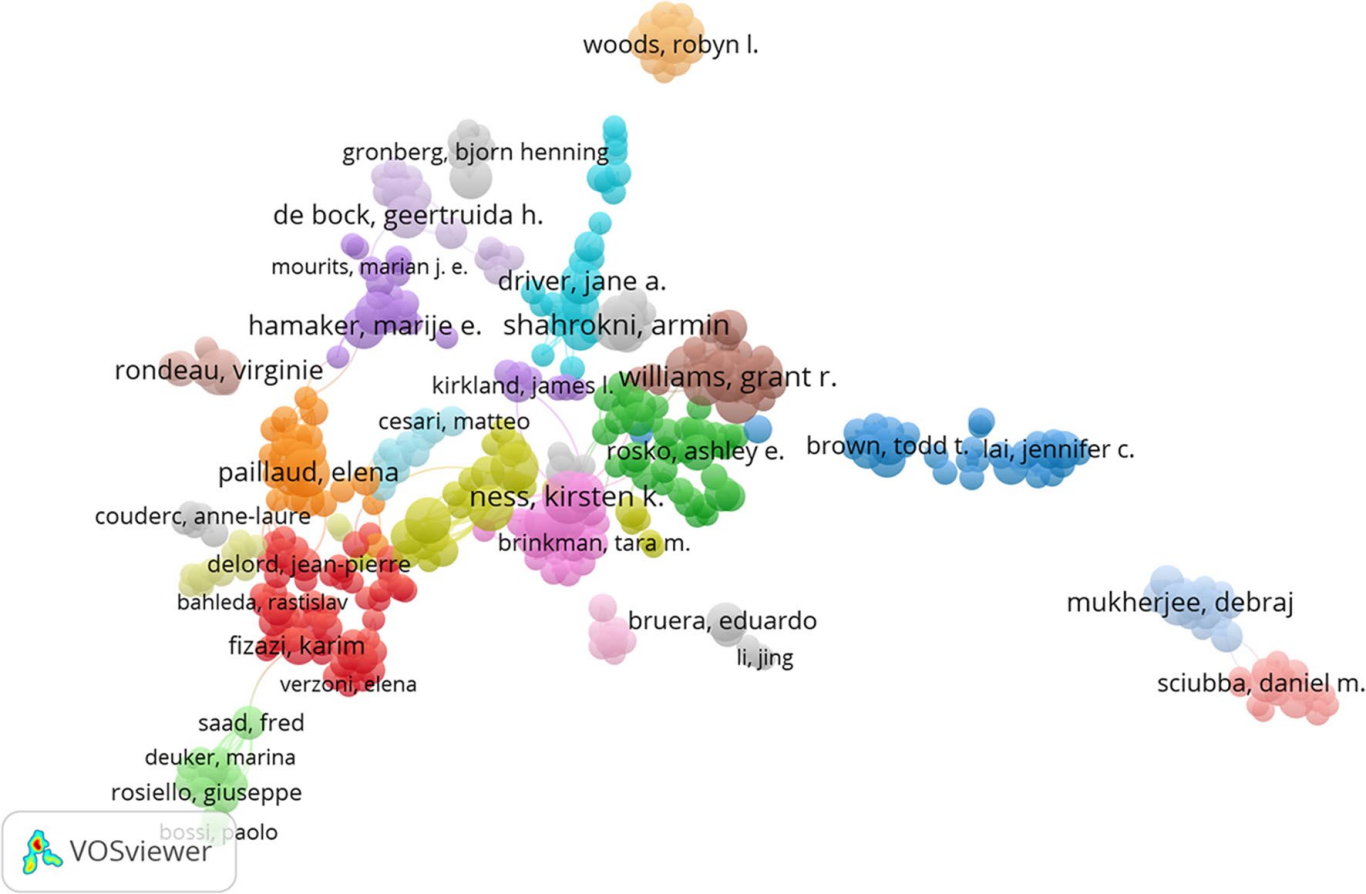
Map of collaborative relationships between authors

**Fig. 8:**
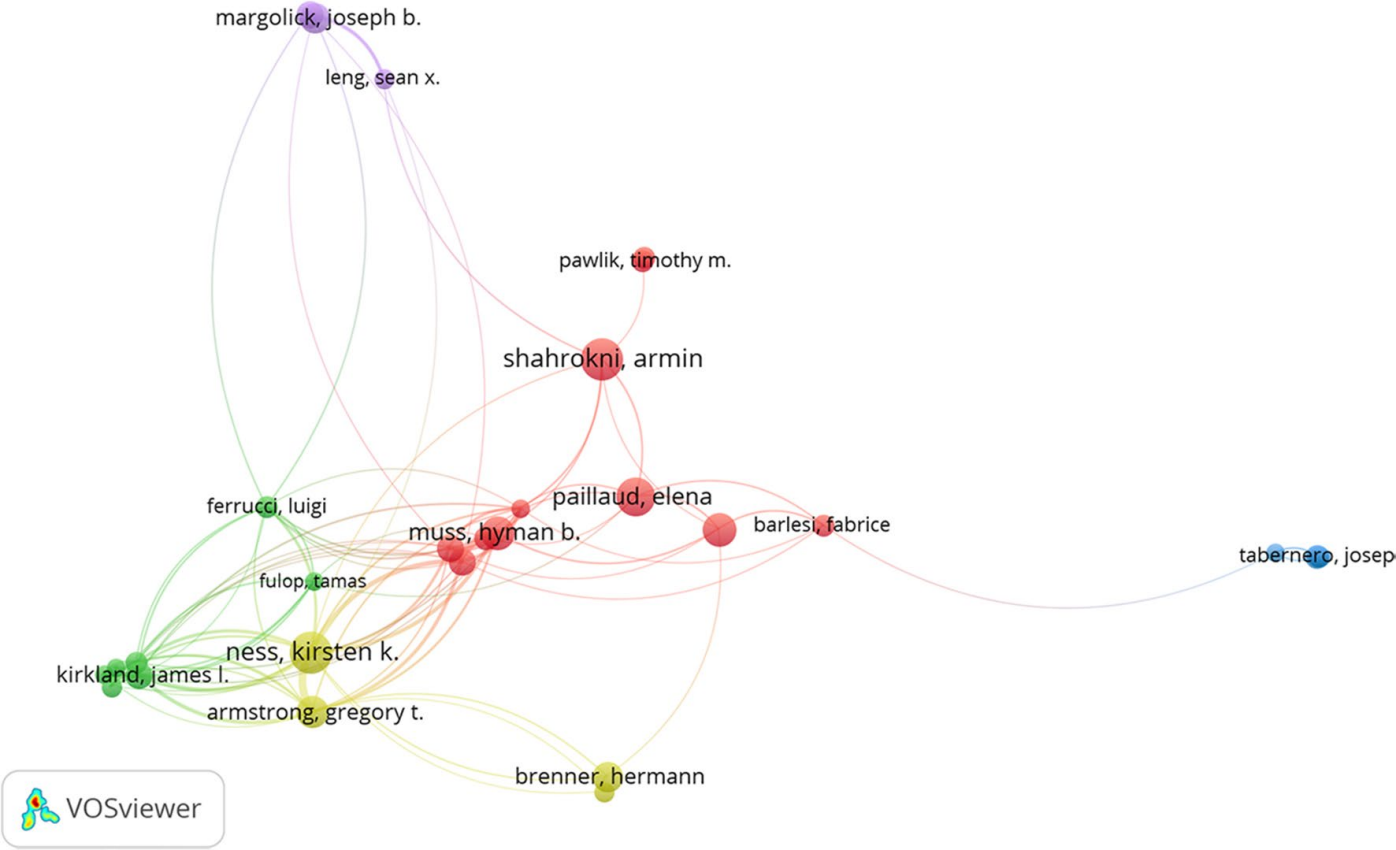
Map of cited relationships between authors

Tables 4 and 5 display detailed information on the most productive and influential authors in the frailty-neoplasms field, including publication counts and citation times, respectively. Kirsten K Ness, with 27 publications, was the field’s most productive author, while James L Kirkland, cited 2146 times, was the most cited author. Figure 7 visualizes the collaboration relationships among authors. The author’s relationship map was segmented into 8 clusters, with sparse relationships between them. Figure 8 illustrates the citation relationships between authors, indicating narrow cited connections.

**Table 4:** The top 10 most productive authors focusing on the relationship between frailty and neoplasms

Author	Documents	Citations	TLS
Ness, Kirsten K	27	953	169
Shahrokni, Armin	26	548	51
Williams, Grant R	25	395	109
Paillaud, Elena	22	524	108
Alibhai, Shabbir M. H	22	332	26
Robison, Leslie L	21	469	147
Kenis, Cindy	21	428	90
Rondeau, Virginie	21	336	16
Wildiers, Hans	20	490	92
Hudson, Melissa M	19	376	142

**Table 5:** The top 10 most influential authors focusing on the relationship between frailty and neoplasms

Author	Documents	Citations	TLS
Kirkland, James L	11	2146	27
Tchkonia, Tamara	8	1864	19
Ferrucci, Luigi	5	1730	12
Oza, Amit M	5	1579	6
Kim, Sung-Bae	5	1321	5
Zhu, Yi	5	1317	13
Robbins, Paul D	5	1243	9
Pignata, Sandro	5	1227	5
Colombo, Nicoletta	7	1213	8
Patel, Manish R	5	1099	4

### Analysis of influential studies

**Fig. 9:**
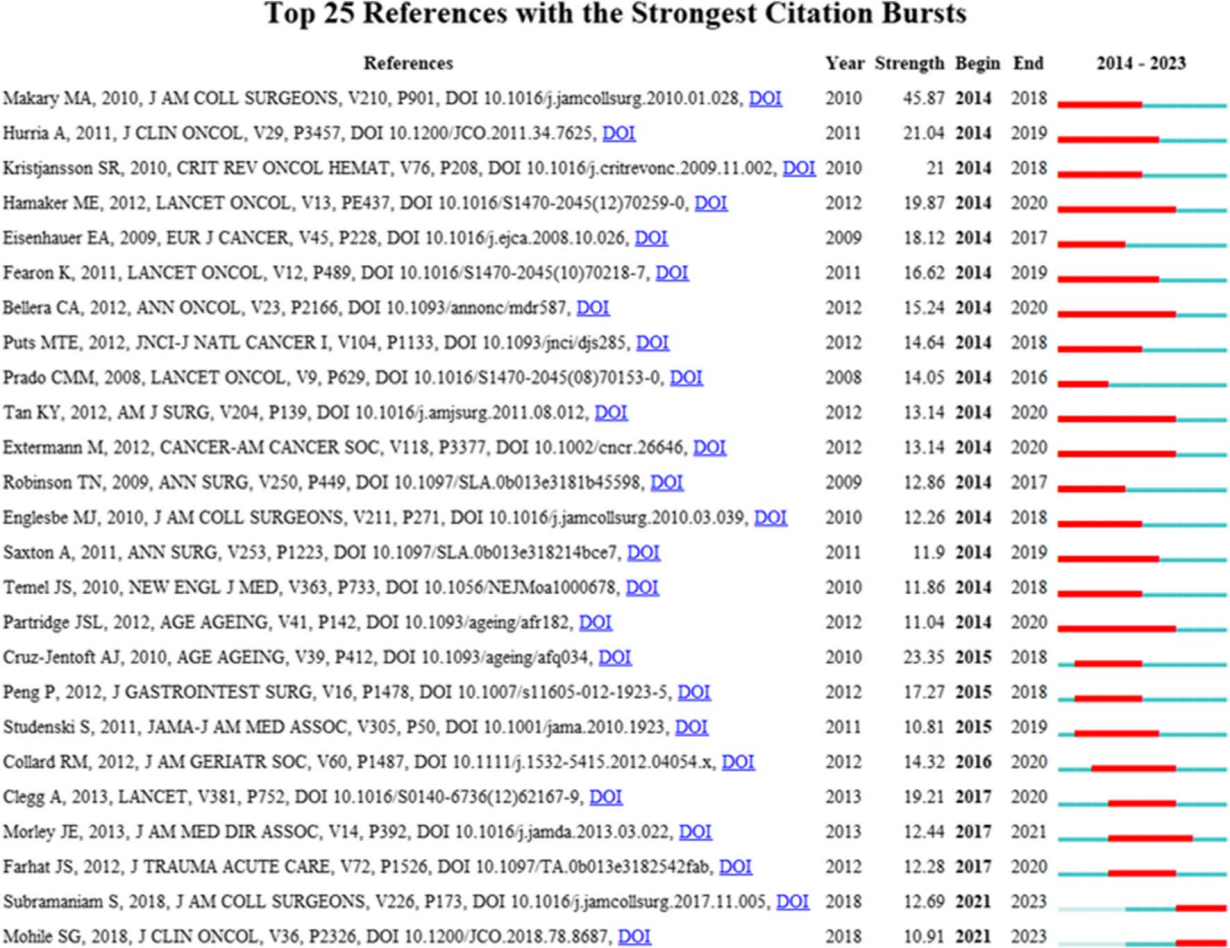
Map of top 25 references with the strongest citation bursts

Table 6 lists the top 10 most-cited publications. All were published between 2015 and 2021, each receiving over 630 citations. The article from *Clinical Interventions in Aging*, cited 1732 times, was the most-cited publication. Figure 9 illustrates burst detection within this field’s literature, with time points showing burst characteristics highlighted in red.

**Table 6:** The top 10 studies with the most citations on the relationship between frailty and neoplasms

Document	Citations	Links
Oxidative stress, aging, and diseases	1732	0
Inflammageing: chronic inflammation in aging, cardiovascular disease, and frailty	1422	1
The Achilles’ heel of senescent cells: from transcriptome to senolytic drugs	1266	0
Lenvatinib versus Placebo in Radioiodine-Refractory Thyroid Cancer	1225	1
Olaparib tablets as maintenance therapy in patients with platinum-sensitive, relapsed ovarian cancer and a BRCA1/2 mutation (SOLO2/ENGOT-Ov21): a double-blind, randomised, placebo-controlled, phase 3 trial	1180	0
Melatonin as an antioxidant: under promises but over delivers	1018	0
The side effects of platinum-based chemotherapy drugs: a review for chemists	983	0
Cellular Senescence Promotes Adverse Effects of Chemotherapy and Cancer Relapse	762	0
Immunosenescence and Inflamm-Aging as Two Sides of the Same Coin: Friends or Foes?	692	0
First-line nivolumab plus ipilimumab combined with two cycles of chemotherapy in patients with non-small-cell lung cancer (CheckMate 9LA): an international, randomised, open-label, phase 3 trial	630	0

### Analysis of keywords

**Fig. 10:**
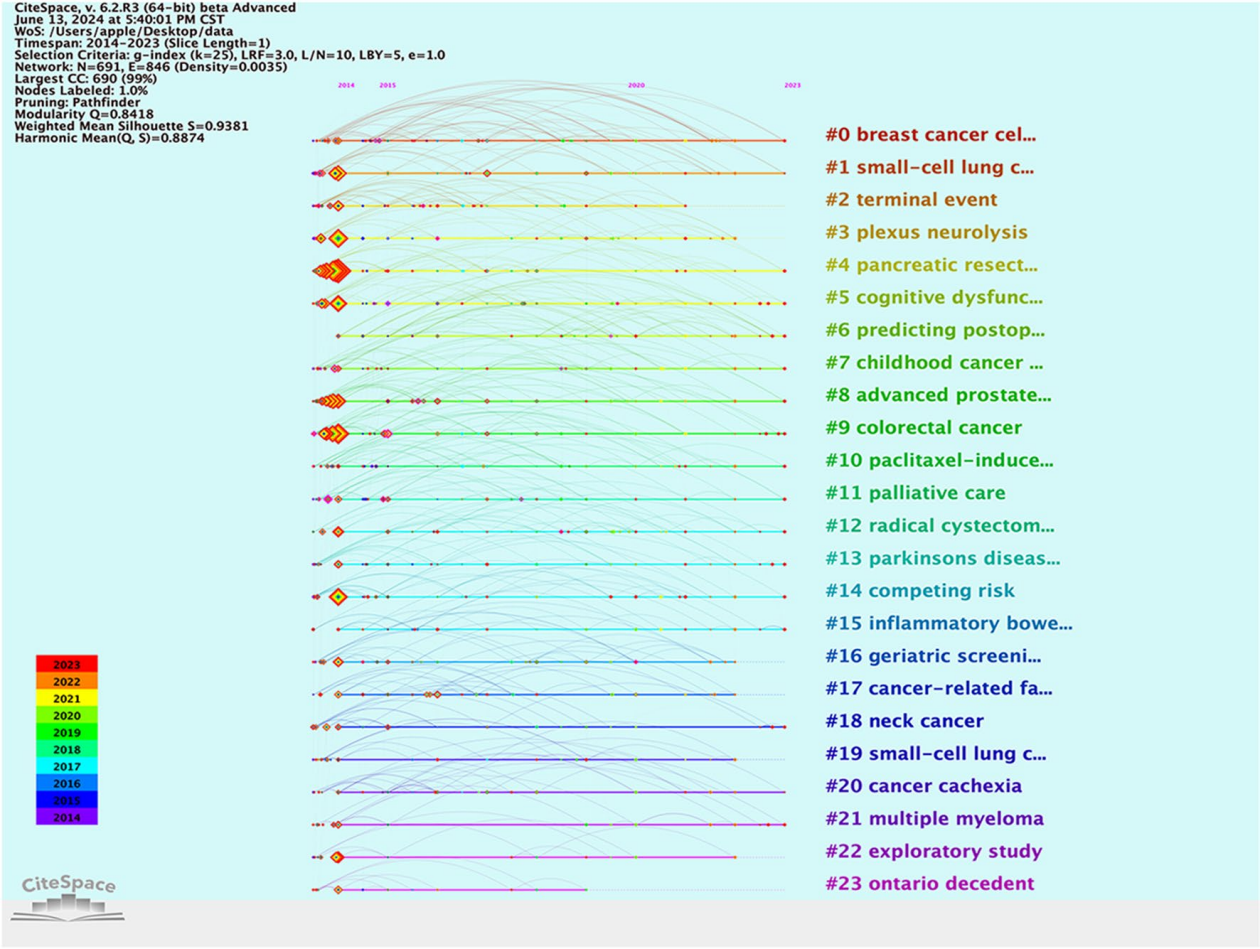
Map of top 25 keywords with the strongest citation bursts

Keywords were extracted from each publication included in our study. Table 7 displays the top 10 most-cited keywords. The five most frequent keywords were “Frailty (1918 occurrences),” “Elderly (1583 occurrences),” “Cancer (1226 occurrences),” “Mortality (864 occurrences),” and “Outcomes (754 occurrences).” Fig. 10 presents the 25 keywords with the highest occurrence intensity. Among these, 23 keywords were from the mutation period concentrated between 2014 and 2019. Keywords with longer duration and higher intensity include “double-blind,” “preoperative assessment,” and “surgical outcome.” During the early to mid-term stages, research focused on observing frailty as an outcome indicator in clinical drug trials, particularly during the perioperative period in tumor patients. This phase emphasized preoperative evaluation and the impact of frailty on postoperative recovery and survival time. Currently, research hotspots focus on the effects of nutrition on tumor patient frailty and nutritional interventions to improve their condition.

**Table 7:** The top 10 keywords with the most occurrences on the relationship between frailty and neoplasms

Keyword	Occurrences	TLS
frailty	1918	3334
elderly	1583	2783
cancer	1226	1527
mortality	864	1778
outcomes	754	1551
survival	704	1167
quality of life	676	783
chemotherapy	572	720
surgery	527	1095
risk	504	898

**Fig. 11:**
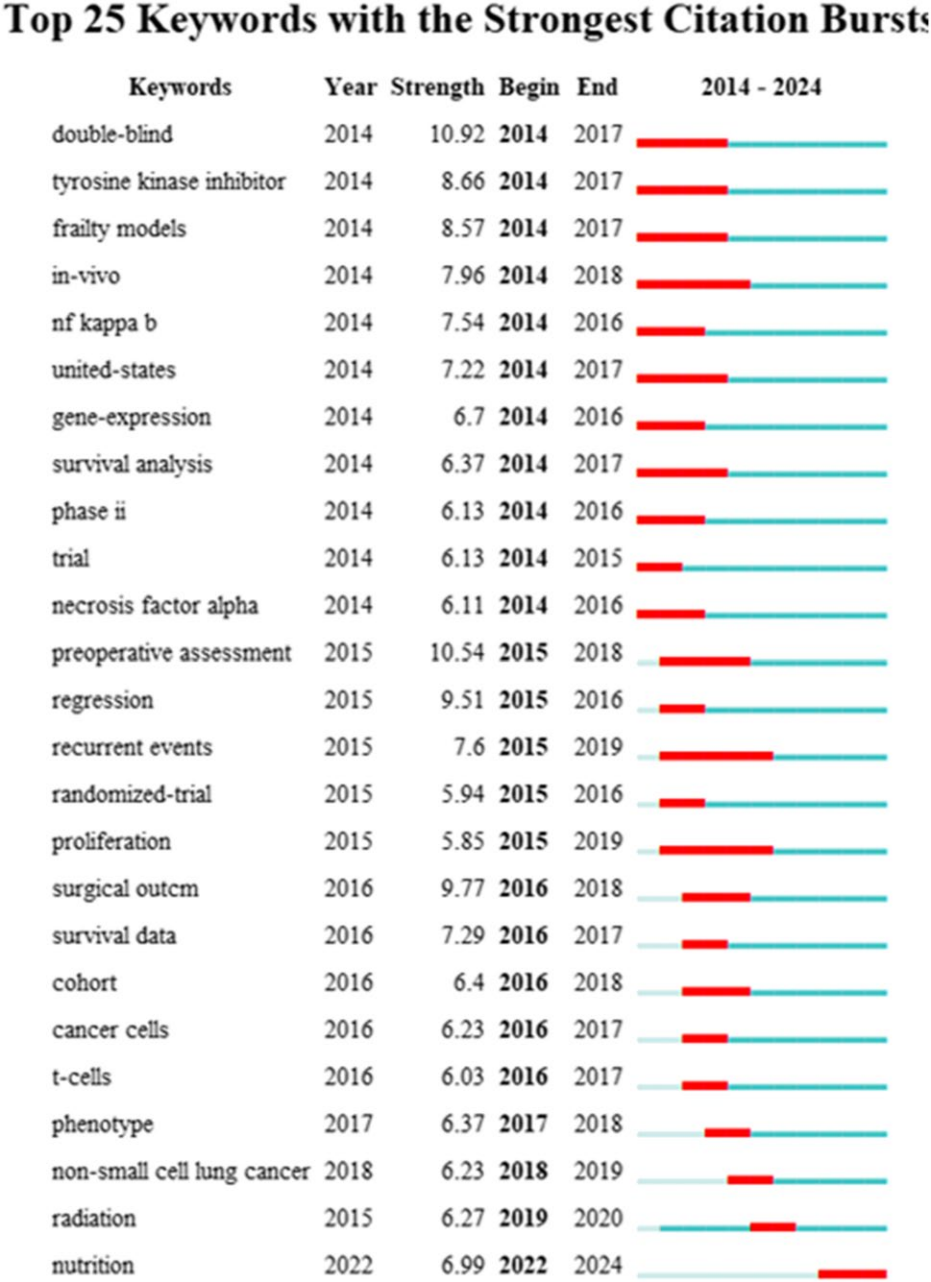
The map of 24 clusters of keywords

A total of 24 clusters were generated from keywords related to tumors and frailty in English literature. The clustering results indicate a *Q* value of 0.8418 and an *S* value of 9381, attesting to the reliability of the clusters [19] (Table 8 and Fig. 11). In the timeline chart, keywords from the same cluster are aligned on the same horizontal line, with the timeline displayed at the top of the chart. The further to the right a keyword is, the more recent it is. Figure 11 clearly illustrates the number of keywords in each cluster and the duration of the research period. A higher number of keywords in a cluster signifies the importance of that research area, while a larger time span indicates the longer duration of that cluster.

**Table 8:** Summary of 24 keyword clusters on the relationship between frailty and neoplasms

Classification	Keyword clusters
Populations and types of diseases	#0 breast cancer cell, #1 small-cell lung cancer, #6 childhood cancer, #8 advanced prostate cancer, #9 colorectal cancer, #13 parkinsons diseases, #15 inflammatory bowel disease, #18 neck cancer, #1 small-cell lung cancer, #21 multiple myeloma, #23 ontario decedent
Clinical manifestations	#5cognitive dysfunction, #20 cancer cachexia
Relevant influencing factors	#3plexus neurolysis, #4pancreatic resection, #10 paclitaxel-inducement, #11 palliative care, #12 radical cystectomy, #14 competing risk,, #17 cancer-related factor
Clinical significance	#16 geriatric screening,#6 predicting postoperative, #22 exploratory study, #2 terminal event

## Discussion

### Current research status on *cancer* frailty

Our study utilized the WoSCC database to gather publication data on the relationship between frailty and neoplasms, taking advantage of its comprehensive and authoritative academic resources [20]. The WoSCC database search results likely provide a comprehensive representation of the current research status in this field. This study indicated that the USA has consistently been the most influential country in advancing research on the relationship between frailty and cancer. The USA not only contributed the most publications on this topic but also received the highest number of citations, underscoring the significance and impact of its research contributions. Additionally, an examination of institutional productivity revealed that nine of the top 10 most productive institutions in this field were located in the USA. The concentration of leading research institutions in the USA highlighted the country’s dominant role in shaping the discourse and progress in frailty and cancer studies. A journal analysis in this field reviewed the top 10 journals with the highest number of publications. Among them, five journals were dedicated to oncology. This distribution underscored the high interest in exploring the relationship between frailty and cancer, as well as the importance of frailty in the field of oncology.

The collective of academically influential authors highlight the trend of research collaboration within a specific discipline. By studying these high-impact authors, we can grasp the hotspots and developments in international frailty research, aiding clinicians and researchers in finding potential collaborators. Our study revealed that the top 10 prolific and highly cited authors predominantly hail from developed countries. Notably, no author possessed both high productivity and citation impact. The top three prolific authors were Ness, Kirsten K. (27 publications, cited 953 times); Shahrokni, Armin (26 publications, cited 548 times); and Williams, Grant R. (25 publications, cited 395 times), with their research primarily focused on clinical interventions and outcome assessments for frailty syndrome [21–24]. The citation rates for these authors were relatively lower. The top three highly cited authors were Kirkland, James L. (11 publications, cited 2146 times); Tchkonia, Tamara (8 publications, cited 1864 times); and Ferrucci, Luigi (5 publications, cited 1730 times). Their research delves into cutting-edge areas such as cellular senescence and biomarkers of aging [25–28]. Although their publication volumes are not among the highest, their work is widely cited due to significant contributions to fundamental science. This highlighted cellular biology and biomarker research as academic hotspots receiving more attention. This analysis indicated that in the field of frailty research, highly cited and prolific authors have different focal points, each playing a unique role in advancing academic development.

### Research hotspots in tumor frailty

In recent years, the relationship between frailty and tumors has been a significant topic of discussion, reflecting the crucial impact of frailty on tumors [29, 30]. Current research explores various aspects of the relationship between frailty and tumors. Scholars focus on frailty’s impact on diseases such as gastrointestinal cancer [31], gynecological tumors [32], multiple myeloma [33], acute lymphoblastic leukemia [34], metastatic cancer [35], and prostate cancer [36]. Some studies indicate that tumors may induce or exacerbate frailty [37]. A single-center study from Brazil involving 179 gastrointestinal cancer patients aged 60 and above found that frailty significantly affected the prognosis of elderly gastric cancer patients undergoing gastrectomy, with frail patients exhibiting poorer outcomes compared to non-frail controls [38]. Multivariable analysis identified frailty as a significant factor influencing postoperative surgical outcomes [39]. Cytokines play a crucial role in the tumor microenvironment. Those associated with lung cancer include interleukin-4 (IL-4), IL-6, monocyte chemotactic protein-1 (MCP-1), and tumor necrosis factor-alpha (TNF-α) [40]. Research shows that frailty can lead to elevated levels of IL-6, C-reactive protein (CRP), and TNF-α in the tumor microenvironment [41]. Therefore, frail patients may exhibit upregulation of cytokine expression, which could result in more malignant tumor cells and enhanced survival capabilities. A meta-analysis revealed that frailty impacts overall survival, all-cause mortality, and chemotherapy toxicity rates in lung cancer patients, and is associated with poorer prognosis [42]. A cohort study involving 4723 cancer survivors showed a positive correlation between the frailty index and all-cause mortality, including cancer and cardiac deaths [43]. Globally, frailty increases with age and is more prevalent in women with breast cancer, underscoring its significance [44]. The prevalence of frailty increases in breast cancer patients following chemotherapy, radiotherapy, and hormone therapy [45].

A 2010 article titled “Frailty as a Predictor of Surgical Outcomes in Older Patients” [46] demonstrated the strongest burst strength, which persisted until 2018. This indicates that surgeons have long been aware of the importance of assessing frailty in elderly cancer patients. Currently, research focuses on improving existing frailty assessment tools to enhance their practicality [47]. Numerous studies emphasize the importance of evaluating frailty in cancer patients, especially during the perioperative period [48]. Increasingly, numerous studies are focusing on improving frailty identification to enhance patient prognosis and the decision-making process [49–51]. A systematic review indicated frailty may be a more accurate predictor of postoperative complications in surgical decision-making than patient age [52]. The study also indicated that, after further validation, the frailty index shows potential in predicting outcomes for patients undergoing brain metastasis surgery. Additionally, a retrospective observational study challenged the notion that age alone is a reliable predictor of frailty or sarcopenia. The study found that individuals in the non-robust group and those at risk of sarcopenia are often physiologically older than their robust, non-sarcopenic peers of the same age [53]. However, it is worth noting that a significant portion of the literature strictly focuses on one or two symptoms of frailty, such as unintentional weight loss and muscle weakness, rather than addressing the overall syndrome of frailty.

### Frontiers and development trends in tumor frailty research

In recent years, the frontier of research on tumors and frailty has increasingly become a significant topic in the field of cancer research. The advent of an aging society has highlighted the importance of studying tumor frailty. Considering the distribution over time and its emergence, research on frailty in tumor patients primarily focuses on the causes and influencing factors of frailty, as well as its assessment. This includes exploring the types of tumors associated with frailty and how the treatment process for tumors affects frailty. Additionally, there is ongoing research into efficient and rapid tools for assessing perioperative frailty in tumor patients. Moreover, efforts have begun to address frailty through interventions in areas such as nutrition. Elderly cancer patients often face high nutritional risks, and the exacerbation of frailty can significantly impact their treatment outcomes. Nutritional supplementation and dietary adjustments can effectively alleviate malnutrition caused by cancer and its treatment, thereby reducing the incidence of frailty [54, 55]. Existing research has established a close relationship between malnutrition and frailty in cancer patients, highlighting that scientific nutritional interventions can significantly improve patients’ frailty, enhance their treatment tolerance, and improve their quality of life. The study of tumor frailty has entered the clinical intervention stage, with nutritional intervention being just the beginning. In the future, proactive interventions for frailty in cancer patients to improve outcome metrics and extend survival time may become a research hotspot.

## Conclusion

Our study focuses on the research trends and status concerning the relationship between frailty and neoplasms over the recent decade, providing valuable insights into the landscape of this important field. Through visual analysis, we found that research in this field is in a phase of rapid development, with a steady increase in the number of related publications. The USA leads in this field. Currently, key research areas include frailty assessment and screening, adverse health outcomes caused by frailty, and studies focused on the elderly and clinical trials. Recently, frailty-related interventions, particularly nutritional interventions, have emerged as a frontier area, representing future development trends.

## Limitation

Citation bias due to publication year is a limitation in this study. Recent publications may naturally receive fewer citations compared to older ones simply due to the shorter amount of time they have been available for citation. This temporal bias can affect the perceived impact or relevance of newer studies within the field, potentially underrepresenting the latest research trends and findings in the analysis. Addressing this limitation in future research could involve adopting a more inclusive approach to conceptual definitions, expanding the range of databases searched, adjusting for publication year in citation analysis, and considering studies published in multiple languages. Such measures would enhance the comprehensiveness and representativeness of the research, providing a more holistic understanding of the intricate relationship between frailty and neoplasms.

## Data Availability

The original contributions presented in the study are included in the article. For supplementary material, further inquiries can be directed to the corresponding authors.
